# *Trichoderma* and Mycosynthesis of Metal Nanoparticles: Role of Their Secondary Metabolites

**DOI:** 10.3390/jof10070443

**Published:** 2024-06-22

**Authors:** Guillermo M. Herrera Pérez, Laura E. Castellano, Claudia A. Ramírez Valdespino

**Affiliations:** 1 Consejo Nacional de Humanidades, Ciencias y Tecnologías (CONAHCYT), Centro de Investigación en Materiales Avanzados, S. C. (CIMAV), Miguel de Cervantes #120, Complejo Industrial Chihuahua, Chihuahua 31136, Chih., Mexico; guillermo.herrera@cimav.edu.mx; 2 División de Ciencias e Ingenierías Campus León, Universidad de Guanajuato, Loma del Bosque #103, Lomas del Campestre, León de los Aldama 37150, Gto., Mexico; laedcato@ugto.mx; 3 Centro de Investigación en Materiales Avanzados, S. C. (CIMAV), Av. Miguel de Cervantes #120, Complejo Industrial Chihuahua, Chihuahua 31136, Chih., Mexico

**Keywords:** green synthesis of nanoparticles, secondary metabolites, *Trichoderma*, mycosynthesis of nanoparticles

## Abstract

Nanocompounds are widely used in many fields such as environmental, medicine, or agriculture. Nowadays, these nanocompounds are mainly synthesized by chemical methods, causing environmental pollution and potential health problems. Thus, microorganisms have been investigated as potential nanoparticle green biosynthesizers. The main research is focused on the synthesis of nanoparticles (NPs) using algae, yeast, bacteria, and fungi. Among them, fungi have been the most used, due to their simple and effective mycosynthesis. Fungi as well as other organisms involved in green synthesis of NPs use their secondary metabolites (SMs) to mediate and catalyze the reactions to produce metal nanoparticles (MNPs) as well as being able to act as capping agents producing different physicochemical characteristics and biological activities in the MNPs. Among the various fungi used for mycosynthesis are *Trichoderma* species, which mediate the production of Ag, Cu, CuO, Zn, ZnO, and other MNPs. Here, we review the main SMs from *Trichoderma* that have been reported or suggested to contribute to synthesize or act as capping agents and their applications, as well as present the main challenges faced by this type of synthesis.

## 1. Introduction

Nanomaterials, specifically MNPs, have peculiar physical and chemical properties not found in bulk metal. These properties are given by their larger surface area compared with their bulk counterparts [1]. The biogenic synthesis of NPs is an attractive nanotechnological alternative to other chemical and physical methods, offering simplicity, relatively low cost, lower generation of toxic waste, lower energy consumption, and higher yields [2]. Plant extracts, algae, yeast, bacteria, and fungi can be used as biofactories of NPs, acting as reducing agents and stabilizers [3]. The ability of such organisms to alter the chemical nature of metals is due to their development of mechanisms of defense against toxic agents, with the products being NPs with lower toxicity [3]. In general, green synthesis mediated by microorganisms is more complex than green synthesis using plant extracts because there are some limitations that make the process longer, including the isolation and cultivation of microorganisms under sterile conditions, as well as their maintenance. However, synthesis mediated by fungi is advantageous compared to plants, because fungi produce a wide quantity of SMs and proteins, which results in a high production of MNPs with longer stability [4].

The use of fungi to synthesize various materials, called mycosynthesis, has gained attention for its potential in various applications, specifically in sustainable agriculture and as antimicrobials, acting against plant and human pathogens [5,6,7,8]. This method has been particularly effective in the production of MNPs, with *Aspergillus*, *Fusarium*, and *Trichoderma* being the main genera for this purpose [9]. Fungi have been found to be effective in the green synthesis of NPs due to their ability to produce large quantities of proteins and SMs, which act as reducing and stabilizing agents [10,11].

Among the fungi listed before, *Trichoderma* is highlighted as an opportunistic avirulent plant symbiont due to its ability to establish mutualistic endophytic relationships with a wide number of plant species including cucumber, tomato, and maize plants; it can control phytopathogens and promote plant defense systems, root growth, and crop productivity [12,13,14,15,16]. By 2020, around 375 species from *Trichoderma* had been reported and validated. They belong to the Fungi Kingdom, Ascomycota Division, Pezizomycoina Subdivision, Sordariomycetes Class, Hypocreales Order, and Hypocraceae Family [17]. These fungi are considered among the most beneficial organisms for humanity, given their versatility to be used in agriculture, biotechnology, bioremediation and, recently, in nanotechnology [18,19]. *Trichoderma* species are of economic importance, due to them being characterized as having rapid growth, as well as their ability to produce of enzymes, antibiotics and other SMs and molecules that help them in their biocontrol activity, and in establishing interactions with plants [20,21].

Secondary metabolites are small molecules that are not necessary for normal growth or development [22]. Some of the SMs are produced by various species when they have ceased to grow and only under certain growth conditions, for example, when the organism responds to certain stresses, giving them the advantage of being able to survive in different environments [23]. Among the main SMs synthesized by fungi are polyketides and fatty acid-derived compounds, aflatoxins, methylsalicylic acid and related compounds, alkaloids, siderophores, quinones and related compounds, terpenes, and peptaibols [23].

It is well known that *Trichoderma* produces a wide variety of SMs with different applications, such as peptaibols, diketopiperazine-type compounds, polyketides, pyrones, and terpenes [24]. These SMs are synthesized through a series of reactions mediated by transferases, p450 monooxygenases, hydrolases, and isomerases, among others, whose genes are generally found in clusters [25]. The plasticity from *Trichoderma* to catabolize a wide variety of substrates as well as produce several SMs makes it a safe and ecofriendly biocontrol agent that is extremely well adapted to different ecological niches, mainly because SMs can act in chemical defense, communication or survive in stress conditions [26]. SMs from *Trichoderma* can also play a key role in reducing toxic metal ions to their metallic forms, producing NPs with different morphological features such as sizes, shapes, and characteristics that can have potential applications in biocatalysis, agriculture, and as antimicrobial agents [5,27]. In this review, we focus on the mycosynthesis mediated by SMs in *Trichoderma.* We also summarize the main SMs reported as key molecules involved in the synthesis and capping of MNPs and their applications and review the main challenges involved in mycosynthesis mediated by *Trichoderma.*

## 2. Mechanism of Green Synthesis of MNPs by Fungi

Compared with physical and chemical methods of synthesis of MNPs, mycosynthesis seems to be simple, but many factors need to be considered to control the size, shape, and stability of the MNPs. Among these factors are the culture conditions to obtain the biomass or the supernatant, the medium composition, temperature, pH, the amount of biomass or supernatant, the precursor and the amount used, and the strain/isolate used, among others [10,28]. 

Two mechanisms have been reported: intracellular and extracellular synthesis. However, most of the reports are hypotheses formulated on the assumption that certain data are obtained, with few or no convincing experimental support. Intracellular synthesis is less common and requires a series of subsequent processes for the purification of the MNPs, which makes it inefficient and cost ineffective. As to how the synthesis is carried out, Mukherjee and collaborators have hypothesized in the case of Ag NPs synthesized by *Verticillium* that Ag^+^ is trapped on the surface of the fungal cells through the electrostatic interaction with negatively charged carboxylate groups in enzymes present there, reducing the Ag^+^ and accumulating on the cytoplasmic membrane as well as within the cytoplasm [29].

**Figure 1:**
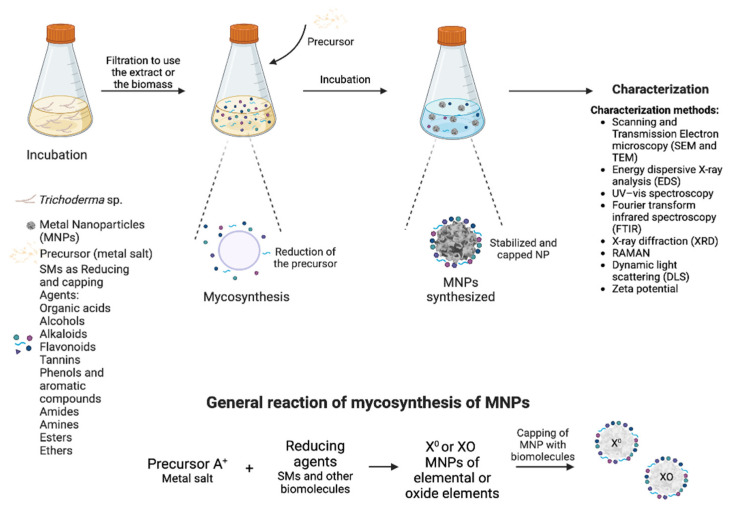
General mechanism of extracellular MNP synthesis mediated by *Trichoderma* SMs. The mechanism of synthesis, methods of characterization and general reaction of formation are schematized.

Extracellular synthesis is the most widely used, as it is simple and cost-effective. As a general methodology for mycosynthesis, the fungus is grown in a nutrient-rich medium for 3–5 days, which can either be used as a source of SMs and other biomolecules, or the biomass is recovered and grown in distilled water for 2–4 days. Then, the supernatant containing the SMs and other biomolecules are recovered and placed in contact with the different precursors, at different concentrations and incubation times. Thus, the different SMs and other biomolecules carry out the reduction of the precursors and, consequently, the precipitation of the MNPs, generally observed as precipitates of different colors, depending on the synthesized NP. Figure 1 shows the general mechanism of how *Trichoderma* SMs are involved in the extracellular synthesis of NPs and the methods used by their characterization.

The extracellular mycosynthesis of MNPs is mediated by reactions with several types of biomolecules, including SMs. The main biomolecules reported are reductases (NADH and nitrate-dependent), quinones, amino acids, organic cofactors, and compounds with glucose, among others [30]. These molecules act by reducing several precursors such as sulfates, acetates, oxides, and chlorides, to obtain the different MNPs.

Some reports indicate that the molecules involved in mycosynthesis are enzymes or enzyme extracts, such as xylanases and amylases from *T. longibrachiatum* L2 and *T. harzianum* MTCC 801, respectively, which showed a potential and new use of enzyme extracts mediating mycosynthesis of Au and Ag NPs [31,32,33]. These results correlate with those previously reported by Gemishev and collaborators where they observed that when growing *Trichoderma* in a medium supplemented with corn steep liquor, the production of Ag NPs was higher compared to the one obtained when *Trichoderma* grew in media with other carbon sources (yeast extract, peptone, and casamino acids) [34]. To assimilate this carbon source, the fungus needs to produce lytic enzymes for its degradation, which are in turn used for the mycosynthesis of MNPs. It is important to highlight that *Trichoderma* is among the fungi with the highest production of lytic enzymes; therefore, those enzymes can be used to synthesize various MNPs with potential use. On the other hand, in *T. viride* and *T. reesei*, it was found that NADH-dependent reductase enzymes are involved in the reduction of Ag^+^ to Ag^0^, in the same way as in *Fusarium* species [34,35,36]. Although these reports indicate that enzymes are responsible for mediating mycosynthesis, this does not exclude the possibility that those other biomolecules, including SMs, could help during the synthesis and capping of MNPs. To corroborate this, one option is to perform the synthesis with the pure enzymes and compare it with a crude enzyme extract and contrast the characteristics of the MNPs. Another alternative is to carry out the deletion of the genes coding for these enzymes and determine if, even in their absence, the synthesis of the MNPs is carried out.

One of the most comprehensive studies on SMs acting by capping MNPs was performed on Se NPs synthesized by *T. harzianum*. By using mass spectrometry analyses via TripleTOF LC-MS, a total of 35 SMs were reported in this study, and 27 of them are antifungal agents. Among these are heptonic acid, ferulate, fumaric acid, threonic acid, mannitol, and glucose [37]. The authors point out that these organic compounds have a dual effect: acting as stabilizers and increasing fungicidal activity. As well, these MNPs had no significant side effects in human cells. These results support the importance of carrying out a careful study of the SMs present in MNPs, to ensure that these may be used in a safe manner, testing on cell lines or other organisms, such as mice, invertebrates, or beneficial plant microorganisms (including *Trichoderma* itself). In the following, we will focus on the SMs described during *Trichoderma*-mediated MNPs synthesis, as well as those that have been suggested for these species.

## 3. Main Secondary Metabolites from *Trichoderma* Involved in the Synthesis of Nanoparticles

Fungal SMs are mainly associated with activities such as competition, mycoparasitism, antibiosis against other microorganisms and predators, induction of microbial growth, communication with plant and other organisms, plant growth regulation, induction of plant resistance, protection against extreme environmental conditions, and as signaling or effector molecules. Thus, the synthesis of MNPs by fungi is a collateral effect resulting from a defense mechanism of the fungus against toxic agents found in the environment.

In mycosynthesis mediated by *Trichoderma*, less is known about what SMs are involved. Most studies report FTIR analysis, which indicates the functional groups present, and mention only that the molecules mediating the synthesis of NPs have organic functional groups in their structure, some of them being SMs [38] (Table 1). 

As mentioned above, depending on the growth conditions of the fungi, they will produce certain SMs. In this regard, it has been reported that pH plays an important role in the production of SMs and, therefore, in the synthesis of MNPs. In Ag NP mycosynthesis, a directly proportional relationship is reported between the size of the NPs and the pH: if the pH is lower, the NPs are smaller. The authors suggest that it was because some fungi at low pH overproduce SMs and other biomolecules that will oxidize the nitrate [39]. On the other hand, it has been reported that Au NPs are unable to be synthesized at low pH, while at neutral and alkaline pH, the formation of MNPs is successfully carried out, probably by protonation of the carboxyl groups present in the SMs [27,40]. In contrast, for the synthesis of Fe NPs mediated by *Trichoderma* species, the optimum pH was found to be 4.5 [41]. It is possible that pH could affect not only the particle synthesis but also the capping and potentially the biological properties, and each presents its unique synthesis parameters.

Interestingly, the use of dead biomass or crude extracts influences the characteristics of the MNPs and their potential applications. do Nascimento and collaborators report the correlation between the biosorption process and the synthesis of Au NPs using biomass of *T. harzianum*, finding that the reduction, nucleation, agglomeration, and capping processes were mediated by molecules with methyl, amide, and amine groups [42]. These results suggest that *Trichoderma* can be used in bioremediation as well as as a biosynthesizer of MNPs. An interesting study that synthesized non-metal NPs used six *Trichoderma* isolates—*T. asperellum*, *T. harzianum*, *T. atroviride*, *T. virens*, *T. longibrachiatum* and *T. brevicompactum*—and evaluated the cultivation method in the form of culture filtrate, cell lysate, and crude cell wall; they found that culture filtrate was more effective and that *T. asperellum* produced the NPs with better results in the promotion of plant growth and in the control of diseases caused by phytopathogenic fungi [43]. These results suggest the versatility of *Trichoderma* in carrying out the mycosynthesis of MNPs, which influences the molecules anchored to them, giving them unique characteristics. Furthermore, these studies indicate that, depending on the MNPs to be synthesized and the *Trichoderma* strain to be used, conditions such as pH and temperature, among others, will have to be standardized to obtain MNPs with the desired characteristics.

One of the possible SMs that could be mediating the synthesis of MNPs are the naphthoquinones and anthraquinones, since it has been reported that these compounds have good reducing properties and these can act as mediators of the synthesis [44].

In the synthesis of Ag NPs, it has been reported that the main SMs involved are alkane, alkene, amine, amide, and organic acid compounds [30,45,46,47,48,49,50]. Interestingly, the synthesis of Ag NPs is one of the most studied, and this has led to the identification of some of the compounds involved in their synthesis. In *T. harzianum*, it was reported that kojic, acetic, and citric acid are the main components mediating the synthesis of MNPs [47]. Another study, also using *T. harzianum*, indicates that the compounds 1-benzoyl-3-[(S)-((2DS, 4R, 8R)-8-ethylquinuclidin-2-yl] (6-methoxyquinolin-4-yl)methyl)thiourea, puerarin, genistein, isotalathiazidine and ginsenoside are responsible for Ag NP production [48]. The first study reports organic acids and the second includes nitrogenous compounds, isoflavonoids and alkaloids, among others. This indicates that, despite using the same *Trichoderma* species, it can produce different SMs and, therefore, mediates the synthesis of the same type of MNPs.

For the synthesis of Au- and Cu-based NPs, SMs of nitrogenous nature stand out [42,47,48,51]. Finally, carboxylic acids, amide, ester, ether, and phenolic compounds mediate the synthesis of ZnO NPs [52]. This information suggests that, depending on the type of MNPs, it will be the nature of the SMs that *Trichoderma* will be using. However, further characterization is needed to determine which SMs are specifically responsible for mediating the synthesis, or at least to suggest candidates containing the identified functional groups.

**Table 1:** SMs involved or suggested in the biosynthesis of *Trichoderma* MNPs.

MNPs	SMs Involved or Suggested to Be Involved in the Biosynthesis	Type of NP/Size of the NP (nm)	Methods of Characterization	*Trichoderma* Species	References
Ag	55 exometabolites: alkane, dicarboxylic acid, aromatic ketone, amino acid, heteroacyclic compound, ketose sugar, sugars alcohol, aliphatic amine, polyol compound, steroidal pheromone, carbocyclic sugars groups	Spherical/ 59.66	SEM, EDAX, Zeta potential, PSA, UV–vis and FTIR	*Trichoderma* fusant Fu21	[30]
Ag	Compounds with –OH group of phenols, alkyne groups, N-H of amine groups, –CH_3_ of aromatic and aliphatic compounds and –C–O stretch of alcohols, carboxylic acids, and esters	Spherical or polyhedral/ 5–50	Electron microscopy, EDS, UV–vis, FTIR and XRD	*T. longibranchiatum*	[45]
	Amide I and amide II-like compounds and primary amines	Spherical/10	UV–vis, TEM, FTIR, DLS and Zeta potential	*T. longibranchiatum*	[46]
	Kojic, acetic and citric acid	Spherical/20	UV–vis and TEM	*T. harzianum*	[47]
	1-benzoyl-3-[(S)-((2DS, 4R, 8R)-8-ethylquinuclidin-2-yl] (6-methoxyquinolin-4-yl)methyl)thiourea, puerarin, genistein, isotalatizidine and ginsenoside	Not determined/21.49	UV–vis, FTIR, EDS, DLS, XRD and SEM	*T. harzianum*	[48]
	Alkaloids, flavonoids, tannins, and phenols	Roughly spherical/ 12.7	XRD, TEM, SEM, EDX and FTIR	*T. harzianum*	[49]
	Biomolecules with hydroxyl, alkane, amide, and carboxylate groups	Spherical, triangular and cuboid/5 to 11	DLS, XRD, FTIR, FESEM and HRTEM	*T. longibrachiatum*	[50]
	Compounds with primary and secondary amines	Triangular and spherical/ 50–75	UV–vis, XRD, FTIR, SEM and EDAX	*T. atroviride*	[51]
	Biochemicals with carbonyl, CH_3_ and alcohol groups	Spherical/ 15–20	UV–vis, FTIR and SEM	*T. viride* and *T. longibraciatum*	[53]
	Biomolecules with amine, amide, carbonyl, phenols, methylene, and alcohol groups	Roughly spherical/5–35	UV–vis, XRD, TEM and FTIR	*Trichoderma* strains	[54]
	Alkaline, amine and aromatic peptides	Anisotropic structural/ 15–25	FTIR, TEM, EDX	*T. atroviride*	[55]
	Secondary metabolites with aromatic, amide I and carbonyl groups	Spherical/ 50–100	UV–vis, FTIR, SEM, EDX, XRD and Zeta potential	*T. citrinoviride*	[56]
Au	Molecules with methyl, amide, and amine groups	Spherical, hexagonal, and octagonal/ 20–50	XRD, FTIR, SEM-EDS, DLS and Zeta potential	*T. harzianum*	[42]
	Compounds with primary and secondary amines	Triangular nanoplates and spherical/ 50–75	UV–vis, XRD, FTIR, SEM and EDAX	*T. atroviride*	[51]
Cu/CuO	Secondary metabolites with amide and aromatic groups	Spherical/110	FTIR, XRD, SEM, TEM and XPS	*T. asperellum*	[57]
	Primary amines, secondary amines, aliphatic amines, and amide groups	Spherical/ 8–100	UV-vis, FTIR and SEM	*T. virens*	[58]
ZnO	Secondary metabolites with carboxylic acid, amide, esters, ethers, and phenolic groups	Spherical or polyhedral/15–30.32	UV-vis, FTIR, EDX, XRD, SEM and TEM	*T. longibranchiatum*	[52]

Abbreviations: Energy dispersive X-ray analysis (EDS); UV–vis spectroscopy (UV-vis); Fourier transform infrared spectroscopy (FTIR); X-ray diffraction (XRD); Transmission electron microscopy (TEM); Dynamic light scattering (DLS); Scanning electron microscopy (SEM); Energy dispersive X-ray (EDAX); Particle size analyzer (PSA); Field emission scanning electron microscope (FESEM); High-resolution transmission electron microscope (HRTEM); X-ray photoelectron spectroscopy (XPS); Raman spectroscopy (Raman) and Photoluminescence (PL).

## 4. Secondary Metabolites from *Trichoderma* Acting as Capping Agents in MNPs

Capping agent is an amphiphilic molecule comprising a polar head group and a non-polar hydrocarbon tail, conferring the functionality and enhancing the compatibility with another phase [59]. Among the molecules reported as stabilizers are amide groups with carbonyl stretch providing stability by enclosing their surface. Thus, secondary metabolites including quinone, alcohol, and organic acids compounds, among others, can serve as stabilizing agents [38,60]. 

The authors reported FTIR analysis where they identified biomolecules that are bound on the MNPs with functional groups such as alcohol, phenols, carbonyl, aromatic and aliphatic amines and amide are presented in the MNPs synthesized [61,62]. However, only a few reports suggest the main SMs involved in mycosynthesis mediated by *Trichoderma*; Table 2 summarizes those that have been described.

Some reports indicate that MNPs were relatively stable up to 6 months after synthesis [46]. Since some molecules involved in capping are SMs, it is important to note that, to maintain their nature, the MNPs must be maintained in a cool and dark environment, otherwise the molecules may be degraded, and the MNPs would lose their stability.

Capping is also responsible for the MNPs having a better antimicrobial capacity; this may be attributable to the biomolecules released by *Trichoderma* that exhibit diverse activities, including antimicrobial activity. Several reports indicate that mycosynthesized MNPs show better antimicrobial activity compared to MNPs synthesized by physical or chemical methods or even some commercial pesticides. In addition, several MNPs exhibit antimicrobial activity similar to those reported for certain commonly used antibiotics [63]. Moreover, an interesting study revealed that TiO_2_ NPs synthesized by *T. citrinoviride* were effective in inhibiting *Pseudomonas aeruginosa*, a drug-resistant bacteria that causes nosocomial infections, and the authors suggest that these MNPs can become an alternative to antibiotics or potentiate the ailing antibiotics [64]. Recently, the mycosynthesis of Ag and Au NPs mediated by *T. saturnisporum* was reported, finding that Ag NPs were more effective against pathogenic bacteria [65], suggesting that, despite being mycosynthesized by the same *Trichoderma*, MNPs from different materials will still have distinctive characteristics and probably different molecules acting as capping. 

In addition, Ag NPs synthesized by *T. harzianum* were shown to be effective for controlling larvae and pupae of the dengue vector *Aedes aegypti* L., suggesting that presumably part of the observable effect is caused by the presence of SMs in the MNPs [63]. Chinnaperumal and collaborators found that TiO_2_ NPs synthesized by *T. viride* had higher larvicidal and pupicidal activity against *Helicoverpa armigera* compared to chemically synthesized TiO_2_ NPs [66]. These results suggest that MNPs synthesized by *Trichoderma* contain SMs capable of controlling organisms of medical and agricultural interest, even better than MNPs obtained by conventional methods.

Mycogenic MNPs produced by some *Trichoderma* species have shown the ability to inhibit the growth of plant pathogenic fungi or have been used in the nanopriming of seeds. Guilger and collaborators reported that Ag NPs synthesized using *T. harzianum* had a major activity against the fungus *S. sclerotiorum* compared to commercial or non-biogenic and uncapped Ag NPs. The authors suggest that this increase is because of the biomolecules and organic compounds present in the capping of the nanoparticles [67]. By using ZnO NPs synthesized by *T. harzianum*, Zaki and coworkers reported that these MNPs showed antifungal activity similar to that reported for two fungicides when evaluated against *Fusarium* sp., *Rhizoctonia solani*, and *Macrophomina phaseolina* [68]. 

Ag NPs were more effective in suppressing disease and improving life on cotton plants, compared with commercial chemical fungicides [69], probably because of the synergistic effect of the MNPs and the SMs contained in them. By using *T. citrinoviride*, TiO_2_ and Ag NPs were synthesized, and it was observed that TiO_2_ at 25 and 50 μg/mL had a positive effect on seed germination and seedling vigor. However, lower concentrations of Ag NPs reduced seed germination and seedling vigor and induced an increase in the activities of catalase, superoxide dismutase, and peroxidase enzymes [56]. The authors suggest that these results, among several factors, are related to the surface charge of MNPs, affecting the absorption and response of seedlings. Recently, MnO NPs were synthesized for the first time by using *T. virens* and they were effective against several phytopathogens, including *Alternaria alternata* and *Helminthosporium* sp. [70].

In addition, the MNPs synthesized by *Trichoderma* can be used as carriers of molecules or in photothermolysis, having potential medical applications [57,71]. Moreover, it has been seen that MNPs synthesized by *Trichoderma* can promote its own growth; this has been reported with FeO_3_ and TiO_2_ NPs and enhances their biocontrol activity [72,73].

One interesting study characterized the removal of contaminants by using MNPs synthesized by *Trichoderma*. The report indicates that SiO_2_ NPs synthesized from rice husks and *T. harzianum* were able to adsorb lead from water used for Nile tilapia culture, and the fish showed increased growth and improvement in physiological parameters; the authors confirmed the presence of functional groups of biomolecules encapsulating SiO_2_ NPs [74]. In these phenomena of adsorption, it is likely that the biomolecules capping the MNPs serve for better adsorption of contaminants; however, no supporting evidence was provided in this study, so further research is needed in this area.

**Table 2:** Secondary metabolites involved or suggested in the capping of MNPs synthesized by *Trichoderma*.

MNPs	SMs Involved or Suggested to Act as Capping	Type of NP/Size of the NP (nm)	*Trichoderma* Species	References
Ag	Exometabolites: alkanes, aromatic alcohol, ketones, phenolic compounds, saturated fatty acids, furans, heterocyclen, steroid, sugar acids, acyclic alkanes, fatty alcohol, aromatic hydrocarbons, esters, and sulfur-containing compounds	Spherical/59.66	*Trichoderma* fusant Fu21	[30]
	Aromatic secondary metabolites	Not determined/21.49	*T. harzianum*	[48]
	Biomolecules with hydroxyl, alkane, amide, and carboxylate groups	Spherical, triangular and cuboid/5–11	*T. longibrachiatum*	[50]
	Compounds with primary and secondary amines	Triangular nanoplates and spherical/50–75	*T. atroviride*	[51]
	Biochemicals with carbonyl, CH_3_ and alcohol groups	Spherical/15–20	*T. viride* and *T. longibraciatum*	[53]
	Biomolecules with amine, amide, carbonyl, phenols, methylene, and alcohol groups	Roughly spherical/5–35	*Trichoderma* strains	[54]
	Secondary metabolites with aromatic, amide I and carbonyl groups	Spherical/50–100	*T. citrinoviride*	[56]
	Compounds with alkane, phosphine, amide and aromatic ketones, aliphatic bending, silica, cycloalkane, aromatic mono-substitution and alkynes functional groups	Spherical/43.68	*T. harzianum*	[62]
	Carbohydrate, and heterocyclic compound molecules, especially, gliotoxin molecule	Spherical and oval/5–50	*T. virens*	[75]
Au	Biomolecules containing amide I and amide II groups	Spherical and pseudo-spherical/9.8	*Trichoderma* sp.	[40]
	Molecules with methyl, amide, and amine groups	Spherical, hexagonal, and octagonal/20–50	*T. harzianum*	[42]
	Compounds with primary and secondary amines	Triangular nanoplates and spherical/50–75	*T. atroviride*	[51]
Cu/CuO	Secondary metabolites with amide and aromatic groups	Spherical/110	*T. asperellum*	[57]
	Primary amines, secondary amines, aliphatic amines, and amide groups	Spherical/8–100	*T. virens*	[58]
MnO	Phenols, alkaloids, carbohydrates, and amino acids	Rod/35	*T. virens*	[70]
Fe/FeO_3_	Compounds with alkene, carboxyl, and phenol groups	Not determined	*Trichoderma* strains	[41]
	Molecules with amide, alcohol, esters, ethers, and aromatic groups	Spherical/185	*T. harzianum*	[72]
	Compounds with amide I and amide II groups	Spherical/25	*T. asperellum*	[76]
SiO_2_	Molecules with different functional groups of biomolecules	Oval, rod and cubical/89	*T. harzianum*	[74]
TiO_2_	Molecules with carbonyl groups	Triangular, pentagonal, spherical and rod/10–400	*T. citrinoviride*	[64]
	Molecules with different functional groups: alkane, methylene, alkene, amine, and carboxylic acid	Roughly spherical/74.4	*T. viride*	[66]
ZnO	Mycochemicals with phenolic, amino acids, aldehydes, and ketone functional groups	Hexagonal, spherical and rod/8–25	*Trichoderma* sp.	[68]

## 5. Research Gaps and Future Directions in the Mycosynthesis of Nanoparticles Mediated by *Trichoderma* and Their SMs

Fungi produce several SMs that can be classified as mycotoxins; it is important to note that mycosynthesis can also lead to the production of these harmful compounds. Tomah and collaborators suggest that in *T. virens*-mediated mycosynthesis of Ag NPs, the SMs involved in capping are organic acids, amide, amine, phenolic and heterocyclic compound molecules, among others, and some of them display medicinal and biocontrol properties but, unfortunately, have toxic properties in humans, such as gliotoxin molecule [75,77]. This research shows one of the most important gaps in *Trichoderma*-mediated mycosynthesis because it is important to focus on the characterization of the SMs involved in their synthesis and capping, since some may contain compounds that could be toxic to humans or animals. It is important to highlight that most studies focus on synthesizing the MNPs, characterizing them by microscopy, XRD, and FTIR techniques and their possible antimicrobial activity or cytotoxicity; however, few are focused on studying the SMs responsible for mediating the synthesis and generating the capping. Thereon, it is important that *Trichoderma*-mediated mycosynthesis begins to be related to metabolomics and proteomics, using techniques such as LC-MS (Liquid Chromatography coupled to Mass Spectroscopy) or NMR (Nuclear Magnetic Resonance Spectroscopy) to know which molecules are involved in the synthesis and capping of MNPs.

Another of the main challenges in mycosynthesis is the fact that the first stages must be carried out under strict sterile conditions to maintain the purity of the strains used; otherwise, probable contaminations can alter the growth of *Trichoderma*, besides affecting the SMs that would be produced, causing a lack of reproducibility during the synthesis process. This consideration can become less cost effective for this type of synthesis; besides, this challenge is not presented in the chemical or physical synthesis as well as phytosynthesis of MNPs and could affect the cost of the synthesis. An alternative to overcome this disadvantage could be if, as mentioned above, a detailed characterization of the SMs or other molecules involved in *Trichoderma*-mediated MNPs synthesis could be performed to purify these compounds or to generate overexpressing strains of these SMs.

One of the sizeable challenges is the synthesis time, because the growth of *Trichoderma* and the obtaining of the extracts can take more than a week, even up to a month. In chemical synthesis, the MNPs are obtained in several hours. In this regard, as mentioned above, one option is to purify the SMs to make them available for the required synthesis, as long as the SMs remain stable for long periods of time. This disadvantage goes hand in hand with large-scale production, where chemical and phytosynthesis methods show better results. Therefore, if MNPs need to be produced with higher efficiency, chemical methods are the best option. However, if what is required are MNPs where their production is environmentally friendly, green synthesis, among them, *Trichoderma*-mediated MNPs synthesis, is one of the best options. One of the research areas to be developed in the following years is the efficiency of green synthesis of MNPs.

On the other hand, most of the fungi reported as nanosynthesizing agents are pathogenic to plants or humans, which makes the disposal of the generated biomass a challenge. In contrast, *Trichoderma* is not a plant pathogen and there are few reports where it has caused infection in immunosuppressed patients. This is an advantage, as only SMs or other biomolecules secreted by *Trichoderma* are used; the biomass produced would be considered a waste, which can be used for the formulation of a biocontroller or biostimulant that can be accompanied by the synthesized NPs and develop other types of agrochemicals, such as bio-nanoformulations with multiple benefits in agriculture. 

To our knowledge, once the MNPs are synthesized by *Trichoderma* and summing up what was found in the cited literature, practically no residues are generated, since, in some syntheses, the MNPs are allowed to be dispersed in the same solution in which the synthesis was carried out and, on the other hand, in other methods, the remains of the extracts are calcined to obtain the desired MNPs. This is a great advantage over chemical methods, where a large amount of highly polluting secondary compounds is generated and for which a special disposal must be carried out. Moreover, the use of toxic and hazardous substances, and the addition of external reducing, stabilizing and capping substances is avoided in mycosynthesis.

Another area of potential interest is to determine the mechanisms of synthesis. An alternative is to generate knockout strains of genes that encode for enzymes involved in the synthesis of a potential SM involved in the synthesis of NPs; in this way, it can be known which SMs are essential for the synthesis to be carried out, as well as which others are important in the capping process. Another alternative is to use computational chemistry tools that allow the theoretical elucidation of which SMs are more feasible to be used in the synthesis and capping process. 

As mentioned throughout this document, the MNPs synthesized by *Trichoderma* can be used in the medical area, as an antimicrobial agent; in agriculture, as an antifungal and pupicidal agent, as well as a stimulant of seed germination and plant growth; likewise, they can be used in remediation processes, by removing toxic agents from the environment, showing even better results than chemically synthesized MNPs. Although, in general, *Trichoderma* mycosynthesized MNPs offer more advantages than disadvantages, there are still areas of study to complement what is currently known and to fully elucidate the mechanisms of synthesis and capping, as well as beneficial or toxic activities for different organisms.

## 6. Conclusions

*Trichoderma* is an alternative for the synthesis of stable MNPs, with unique characteristics, and although *Trichoderma*-mediated mycosynthesis has been investigated for about 20 years, little is known about the SMs involved in this process. Therefore, it is a field of research with high potential that could lead to the search or generation of new strains with higher production of these SMs. Recently, more than 70 strains from *Trichoderma* were reported and they produce around 445 new metabolites, including terpenes, steroids, polyketides, peptides, and alkaloids, which can be used as reducing or capping agents [78].

However, it is important to conduct detailed studies to understand the biochemical mechanism that allows *Trichoderma*-mediated MNP synthesis to be carried out, as well as the composition of the *Trichoderma* SMs involved in the synthesis and capping of MNPs to ensure that their use will not generate undesirable side effects that could impact both animal health and ecosystems. This opens a new line of research, which will establish the guidelines to produce MNPs in a more sustainable and safe manner.
